# Effects of pulsed electromagnetic field (PEMF) on the tensile biomechanical properties of diabetic wounds at different phases of healing

**DOI:** 10.1371/journal.pone.0191074

**Published:** 2018-01-11

**Authors:** Harry M. C. Choi, Alex K. K. Cheing, Gabriel Y. F. Ng, Gladys L. Y. Cheing

**Affiliations:** Department of Rehabilitation Sciences, Faculty of Health and Social Sciences, The Hong Kong Polytechnic University, Hung Hom, Kowloon, Hong Kong; Illinois Institute of Technology, UNITED STATES

## Abstract

The present study investigated the effects of pulsed electromagnetic field (PEMF) on the tensile biomechanical properties of diabetic wounds at different phases of healing. Two intensities of PEMF were adopted for comparison.

We randomly assigned 111 10-week-old male streptozotocin-induced diabetic Sprague-Dawley rats to two PEMF groups and a sham control group. Six-millimetre biopsy punched full thickness wounds were made on the lateral side of their hindlimbs. The PEMF groups received active PEMF delivered at 25 Hz with intensity of either 2 mT or 10 mT daily, while the sham group was handled in a similar way except they were not exposed to PEMF. Wound tissues were harvested for tensile testing on post-wounding days 3, 5, 7, 10, 14 and 21. Maximum load, maximum stress, energy absorption capacity, Young’s modulus and thickness of wound tissue were measured.

On post-wounding day 5, the PEMF group that received 10-mT intensity had significantly increased energy absorption capacity and showed an apparent increase in the maximum load. However, the 10-mT PEMF group demonstrated a decrease in Young’s modulus on day 14. The 10-mT PEMF groups showed a significant increase in the overall thickness of wound tissue whereas the 2-mT group showed a significant decrease in the overall maximum stress of the wounds tissue.

The present findings demonstrated that the PEMF delivered at 10 mT can improve energy absorption capacity of diabetic wounds in the early healing phase. However, PEMF (both 2-mT and 10-mT) seemed to impair the material properties (maximum stress and Young’s modulus) in the remodelling phase. PEMF may be a useful treatment for promoting the recovery of structural properties (maximum load and energy absorption capacity), but it might not be applied at the remodelling phase to avoid impairing the recovery of material properties.

## Introduction

People with diabetes have difficulty in wound healing and medical interventions are required to enhance healing. Without proper glycaemic control, managements of complications and suitable footwear, diabetic foot ulcers will develop and this may lead to chronic ulcers and even amputations which severely decrease the patients’ quality of life [1, 2].

Pulsed electromagnetic field (PEMF) has been used clinically as an intervention to enhance healing of chronic ulcers. Previous studies have shown that PEMF accelerated wound closure [3–6], reduced wound pain [4], enhanced healthy granulation [4] and promoted circulation [6]. A systematic review concluded that PEMF could significantly accelerate the healing of chronic ulcers (decubitus, venous and plantar) in patients [7].

On the other hand, animal studies also provide evidence for the use of PEMF in enhancing wound healing. Generally in non-diabetic models, PEMF reduced wound size [8–11], resolved inflammation [11], accelerated re-epithelialization [11], promoted vascular growth [9, 11], improved fibroblast maturation [11], enhanced collagen deposition [9, 11] and increased the tensile strength [12] of cutaneous wounds.

For diabetic models, there were impairments in wound closure [13, 14], vascular growth [14] and tensile strength of wound [13] as compared to non-diabetic animals. PEMF has been shown to rescue the impairments, and accelerate wound closure [13, 14], promote vascular growth [14], improve blood circulation [14], increase myofibroblast population [15], enhance collagen deposition [16] and increase tensile strength [13] in the diabetic wound models.

Biomechanical properties of wounds are contributed by the underlying structure. For example, tensile biomechanical properties are mainly contributed by the amount of collagen, fibril alignment and fibre orientation. The assessment of biomechanical properties of wounds can reveal the functional outcome of the wound tissue. Recently, our research team has demonstrated that PEMF promotes collagen deposition in diabetic wounds [16]. Although Goudarzi et al. [13] reported that 10-day exposure to PEMF increases the maximum stress of diabetic wounds, they did not report the changes in other tensile biomechanical properties such as maximum load, energy absorption capacity and Young’s modulus over time. Detailed assessment of the tensile biomechanical properties can provide more information on the structural recovery of the wounds and the effects of PEMF. Note that maximum load and energy absorption capacity (structural properties) are contributed by collagen abundance and orientation. However, maximum stress and Young’s modulus (material properties) do not depend on the mass, instead, they are mainly contributed by the quality, orientation and cross-link density of collagen fibres [17, 18]. Measuring the structural and material properties at different phases of wound healing can provide useful information on the effects of PEMF at specific wound healing time frame.

Even though some studies have investigated the effects of PEMF on wound healing, various research groups adopted different PEMF parameters, and this may account for the contradictory findings reported in these studies. For example, two studies [9, 19] adopted very different intensity, frequency, waveform and treatment time of PEMF; Ottani et al. [9] showed that PEMF produced overall improvements on wound closure, neo-vascular growth and collagen deposition, which were contrary to the negative findings reported by Glassman et al. [19]. Pilla et al. [20] proposed that the choice of PEMF parameters are crucial in determining the efficacy of PEMF. In order to evaluate the optimal PEMF treatment protocol in enhancing diabetic wound healing, the present study compared the effects of PEMF delivered at higher (10 mT) and lower (2 mT) intensities on the tensile biomechanical properties of diabetic wounds at different phases of healing. The present study successfully demonstrates that PEMF of the two intensities altered the tensile biomechanical properties of the diabetic wounds differently at various phases of healing.

## Materials and methods

### Animal handling and diabetes induction

The protocol was approved by the Animal Subjects Ethics Sub-Committee of the Hong Kong Polytechnic University (Permit Number: 11/12). Wound induction was performed under ketamine/xylazine anaesthesia. Animals were sacrificed by cervical dislocation under anaesthesia. All efforts were made to minimize suffering. A total of 150 10-week-old male adult (300–400 g) Sprague-Dawley rats were used. All of the rats received humane care and the protocols were in compliance with the guidelines from the Animal Subjects Ethics Sub-Committee. The rats were first housed in groups of two to three under controlled temperature (21°C) and humidity (60%) following 12-hour light-dark period. They were fed with a standard laboratory diet and water *ad libitum*. After 7 days of acclimatization, the rats were fasted for 12 hours before diabetes induction. To induce diabetes, 10 mg/ml of streptozotocin was prepared in a sterile citrate buffer (pH 4.4) and then injected intra-peritoneally (at a dosage of 50 mg/kg rat body weight) in the morning. Just before the injection, the blood glucose level of the rats was measured to reject any abnormal cases. After 7 days, the blood glucose level of the rats was measured again and monitored regularly throughout the experiment to ensure the diabetic condition sustained. Any rats with a blood glucose level lower than 16.7 mmol/L were excluded from the study.

### Wound induction

Before wound induction, mixtures of ketamine and xylazine were administered intra-peritoneally at a dosage of 100 and 3.33 mg/kg body weight respectively. After shaving and disinfecting with betadine and alcohol prep, the wounds were induced with sterile 6-mm biopsy punches on the lateral side of each hindlimb (about 3 mm distal to the fibular head). The wounds were left open and the rats were then housed individually to prevent cannibalism. Photographs of wounds were taken on post-wounding days 0, 3, 5, 7, 10, 14 and 21 and wound area were analysed by the Fiji software [21]. Percentage wound area was calculated by dividing the wound area at the time point with the wound area on day 0, and then multiplied by 100%.

### PEMF treatment

The rats were randomly allocated into sham and active PEMF groups. To examine the effects of high and low intensities, 2-mT and 10-mT PEMF treatment were included. Starting from post-wounding day 1, the PEMF groups were treated with PEMF of 25 Hz and either 2, or 10 mT, 1 hour daily. PEMF was generated by a commercially available device (BTL-4000, BTL Industries Ltd., UK). During PEMF exposure, the rats were restrained in plastic restrainer bags. Their hindlimbs with wounds were placed on the disc applicator of the PEMF device. The sham PEMF group was handled in a similar manner except the device was not activated.

### Biomechanical testing

On post-wounding days 3, 5, 7, 10, 14 and 21, rats were randomly selected, euthanized and then kept frozen at -80°C until the biomechanical testing. The rats were thawed at room temperature for at least 6 hours before testing.

A tensile tester (MTS Synergie 200 machine, MTS Systems Corporation, Eden Prairie, MN, USA) and testing software (TestWorks 4 Universal, MTS Systems Corporation, Eden Prairie, MN, USA) were used to measure structural and material properties of the wound tissue.

The wound beds in a skin layer of 6.0 mm × 18.0 mm were dissected from the bodies of rat along the fibula. The actual length, width and thickness of the extracted tissue were measured with a Vernier calliper. The specimens were elongated to a 2.5% strain position at 0.167 mm/s for 10 preconditioning oscillation cycles [22]. The tensile biomechanical properties were measured by elongating the specimen at a speed of 8.33 mm/s until failure [23]. Since the toughness of the skin at the sole, where diabetic ulcers occur, is critical during walking, we adopted this movement-related high tendon/ligament-testing speed in order to increase the clinical relevance of the study. Load and deformation were recorded at 100 Hz. During the whole testing procedure, the specimens were kept moist. A load-deformation curve was plotted to examine the structural properties and the curve was transformed into a stress-strain curve by dividing the load with the cross-sectional area (stress) and the elongation with the original length (strain) to examine the material properties.

### Statistical analyses

Two-way ANOVA with post-hoc LSD tests were used to examine the group × time interactions and the overall group (sham, 2-mT and 10-mT PEMF) effects. Since Shapiro-Wilk test revealed non-normality of the data, Kruskal-Wallis with post-hoc Bonferroni corrected Mann-Whitney U tests were used to test the effects of groups on the % wound area, biomechanical properties and thickness of the diabetic wounds at different time points. Data were expressed as mean ± standard error of mean (s.e.m.). Significance level was set at 0.05. The analyses were executed by IBM SPSS statistics (IBM Corp. Released 2012. IBM SPSS Statistics for Windows, Version 21.0. Armonk, NY: IBM Corp.).

## Results

Eventually, 33 of the 150 rats died of streptozotocin injection or anaesthesia. Among the 117 surviving rats, 6 failed to develop hyperglycaemia. A total of 111 rats were therefore used for the subsequent experiments. Of the 111 rats, since some wounds were randomly selected for other pilot studies, we finally used a total of 215 wounds in this study. Details of sample size and grouping were summarized in S1 Table.

**Fig 1:**
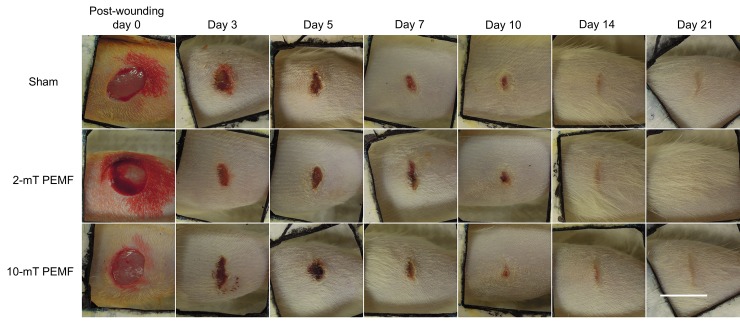
Representative wounds from sham, 2-mT and 10-mT PEMF groups on post-wounding day 0, 3, 5, 7, 10, 14 and 21. The area of wounds of the 2-mT PEMF group was slightly smaller on day 3. All photographs were taken with a 20 mm × 20 mm scale. Scale bar: 10 mm.

**Fig 2:**
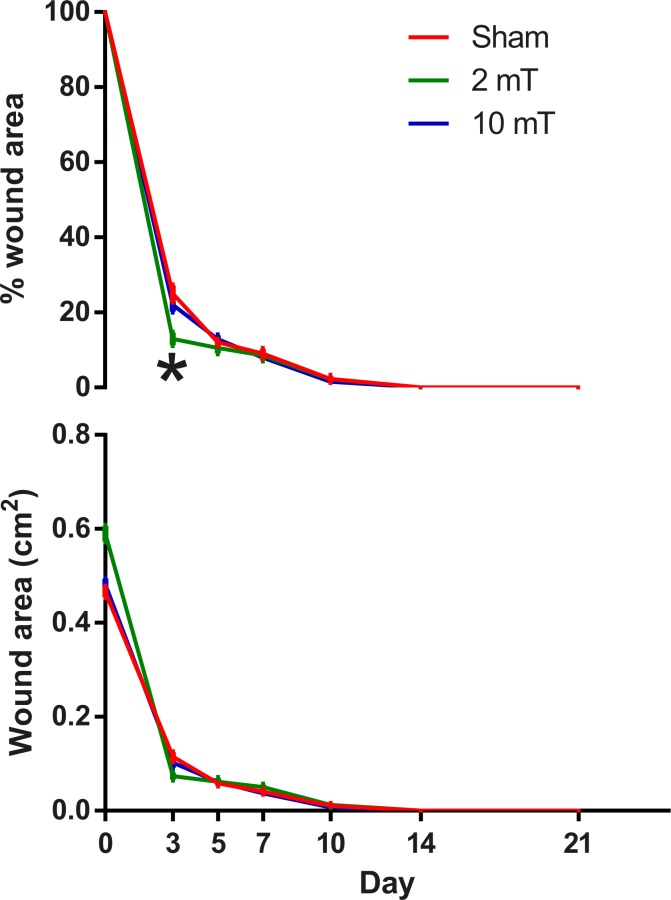
Wound area and percentage wound area measured from sham, 2-mT and 10-mT PEMF groups on post-wounding day 0, 3, 5, 7, 10, 14 and 21. Data are expressed as mean ± s.e.m. The % wound area of the 2-mT PEMF group was significantly smaller than that of the sham PEMF group (p = 0.036). * p < 0.05. The data used to plot this figure can be found in S1 Data.

Throughout the experiment, hyperglycaemia was maintained and the body weight of rats decreased over time despite the increased consumption of water and food. These were typical signs of successful induction and maintenance of diabetic condition in the rats. No signs of wound infection were observed in any of the rats. By day 14, all wounds had grossly closed leaving no scab (Fig 1). Significant overall group effect was found on the % wound area (p = 0.025); the % wound area of the 2-mT group was significantly smaller than that of the sham group (p = 0.02). On day 3, the % wound area of the 2-mT group was significantly smaller than that of the sham PEMF group (Fig 2, p = 0.024).

**Fig 3:**
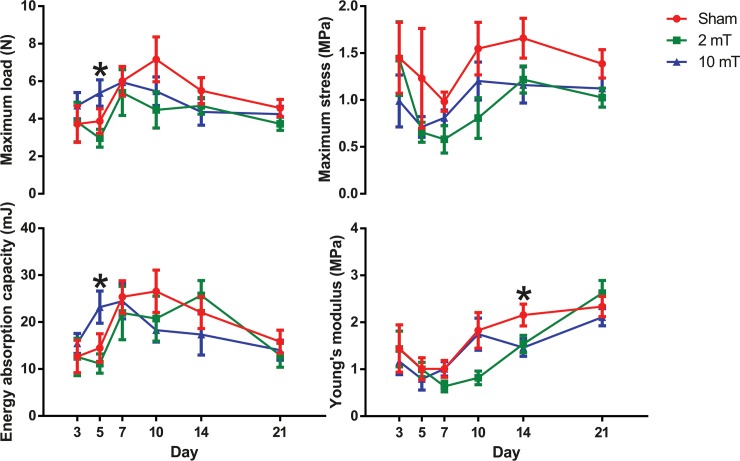
Biomechanical properties measured from sham, 2-mT and 10-mT PEMF groups on post-wounding day 3, 5, 7, 10, 14 and 21. Significant between-group differences were found in maximum load (2-mT vs. 10-mT) and energy absorption capacity (sham vs. 10-mT and 2-mT vs. 10-mT) on day 5, and in Young’s modulus (sham vs. 10-mT) on day 14. Overall, both PEMF groups (2-mT and 10-mT) tended to have smaller maximum stress than the sham group. Data are expressed as mean ± s.e.m. * p < 0.05. The data used to plot this figure can be found in S2 Data.

As for biomechanical properties (Fig 3), there was a significant overall group effect (p = 0.027) that the maximum load of the 10-mT PEMF group was greater than that of the 2-mT group (p = 0.049). On day 5, the maximum load of the 10-mT was also significantly greater than that of the 2-mT (p = 0.012). The energy absorption capacity of 10-mT PEMF group was significantly greater than that of sham (p = 0.036) and that of the 2-mT (p = 0.008) on day 5. There was a significant overall group effect (p = 0.011) that the maximum stress of the 2-mT PEMF group was smaller than that of the sham group (p = 0.015). On day 14, the Young’s modulus of the 10-mT group was significantly smaller than that of sham (p = 0.023).

**Fig 4:**
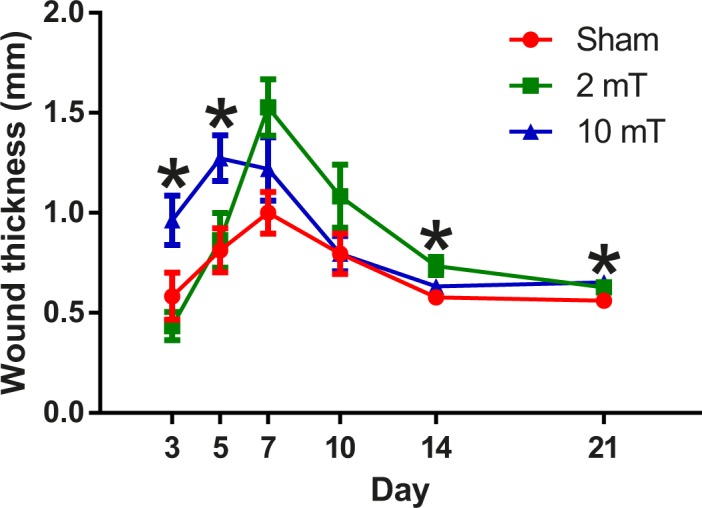
Thickness of wounds measured from sham, 2-mT and 10-mT PEMF groups on post-wounding day 3, 5, 7, 10, 14 and 21. Significant between-group differences were found on day 3, 5, 14 and 21. Overall, the 10-mT PEMF group tended to have greater wound thickness than the sham group. Data are expressed as mean + s.e.m. * p < 0.05. The data used to plot this figure can be found in S2 Data.

Significant overall group effect on wound thickness was found (p = 0.01); the wound thickness of 10-mT PEMF group was significantly greater than that of the sham group (p = 0.007). The wound thickness (Fig 4) of the 10-mT group was significantly greater than that of the 2-mT group (day 3: p = 0.002) and the sham PEMF group (day 5: p = 0.014, day 21: p = 0.022). On day 14, wound thickness of the 2-mT group was significantly greater than that of the sham group (p = 0.004).

## Discussion

This study was the first study that examined the effects of PEMF on various tensile biomechanical properties of diabetic wounds over a period of time. It had lasted for 3 weeks so as to cover the inflammatory, proliferative and part of the remodelling phases of wound healing.

In terms of wound closure, our findings were in line with those reported by other authors [13–15], suggesting that PEMF could enhance wound closure. Our study delivered PEMF at 25 Hz with an intensity of 10 mT, which is comparable to that of 20 Hz and 8 mT adopted by Goudarzi et al. [13]. The lower intensity of 2 mT used is comparable to the 1.2-mT and 15-Hz PEMF adopted by Callaghan et al. [14] and the 5-mT and 25-Hz by our earlier study [15]. The present findings illustrated that PEMF delivered at lower intensity might be more effective in enhancing wound closure, at least in the early phase of diabetic wound healing (Figs 1 and 2). Both Callaghan et al. [14] and Goudarzi et al. [13] found that PEMF could reduce the time for complete closure in diabetic wounds. However, different diabetic wound models were adopted in the two previous studies. Callaghan et al. [14] used a model with minimal wound contraction while Goudarzi et al. [13] used large wounds. In the present study, the effect of PEMF on reducing time for wound closure might be masked by the effects of wound contraction, and also the small wound size. The present study did not adopt wound splinting; the assessment of wound closure might be confounded by the mobile and contractile panniculus carnosus. However, photographs of the wounds in the present study were taken with the hindlimbs placed in a standardized position while the animals were under anaesthesia in order to minimize the inconsistency due to the mobility and contractility of the non-splinted panniculus carnosus. Although wound splinting may solve the technical issue during wound closure assessment and minimize wound contraction, this manipulation may induce external mechanical forces that interact with myofibroblasts, fibroblasts, and collagen synthesis and remodelling, confounding our biomechanical measurements of the wound tissue. This is a dilemma and limitation in wound studies using murine models.

Instead of measuring the strength of wounds at a single time point in the late phase of wound healing, we included different time points in order to examine the different wound healing phases. We found that 10-mT PEMF increased the maximum load and energy absorption capacity in the early phase only. However, after normalizing the maximum load with cross-sectional area as maximum stress, no significant difference was found between groups. This can be explained by the increased thickness in 10-mT group on day 3 and 5 compared to the sham group. These results reflected that 10-mT PEMF could promote proliferation and collagen deposition but not necessarily lead to better quality or alignment of the fibres. In fact, our earlier studies [15, 16] had shown that PEMF increased myofibroblast population and collagen deposition in the early phase of diabetic wounds, but not the collagen quality and alignment. Although 2-mT PEMF did not appear to enhance proliferation and collagen deposition in the early phase, its effects were apparent in the later phase by the time re-epithelialization was completed on day 10. This is supported by the trend of increased wound thickness on day 7, day 10 and significantly day 14, as compared with the sham group. These findings showed that PEMF of different intensities may affect diabetic wound healing differently at a specific time window.

The maximum stress and Young’s modulus are independent to the mass of the wound tissue but the alignment and orientation of collagen [17]. The finding that the PEMF groups had an overall smaller maximum stress and a significant decrease of Young’s modulus than the sham group by day 14 reflected that PEMF may not promote collagen alignment and orientation especially in the late phase after re-epithelialization had completed. This finding was consistent to those reported in our earlier study that PEMF did not have benefits for promoting alignment of the collagen during diabetic wound healing [16]. Moreover, the present study did not support the notion that PEMF could strengthen the diabetic wounds after complete re-epithelialization, which was different from the report by Goudarzi et al. [13], in which they found PEMF had significantly increased the maximum stress. In their study, the PEMF treatment was stopped on day 10, which was just before complete re-epithelialization. Strauch et al. [12] reported PEMF-induced increment in tensile strength, although in non-diabetic wounds. In their study, PEMF treatment was given even after complete re-epithelialization. But the effective intensities used were 100 μT and 5 μT, which are very small as compared to other studies. Therefore, both intensity and treatment period of PEMF appear to be important determining factors for the treatment outcomes.

The present study speculated that 10-mT PEMF can probably augment proliferation and collagen deposition in the early phase of healing that is associated with an increased energy absorption capacity and wound thickness, whereas 2-mT PEMF may enhance proliferation in the later phase. Nonetheless, PEMF of either intensity did not increase the maximum stress and Young’s modulus after complete re-epithelialization. Conversely, the PEMF weakened the wounds in the remodelling phase. This phenomenon may be related to an increase in cell proliferation and collagen deposition but not the fibre orientation, fibril alignment and cross-link density. Limited studies have evaluated the underlying changes in histology and biochemistry of wounds brought by PEMF. Our previous studies [15, 16] showed that PEMF can increase collagen deposition, possibly due to increased proliferation or differentiation of myofibroblast, but not fibre orientation and fibril alignment. Fibroblast growth factor-2 (FGF-2) is one of the growth factors playing critical roles in wound healing. Callaghan et al. [14] found that PEMF increased level of FGF-2 in a mouse diabetic wound model. The increased FGF-2 level could contribute to enhanced cell proliferation and collagen deposition. In line with the collagen deposition and proliferation enhancing effect of PEMF-mediated FGF-2 increase, Tepper et al. [24] reported that PEMF increased endothelial release of FGF-2 *in vitro*, and the PEMF-treated endothelial FGF-2 enriched medium promoted *in vitro* fibroblast proliferation, which is critical for collagen deposition in wound healing. However, PEMF did not directly promote fibroblast proliferation [24]. FGF-2 is known to be a potent stimulator of fibroblast proliferation and collagen production [25–27]. Treatment of recombinant FGF-2 significantly elevated type I collagen expression and cell proliferation in cultured tenocyte, which is a specialized type of fibroblast [25]. Moreover, Madry et al. [26] illustrated that sustained and enhanced FGF-2 expression delivered by recombinant adeno-associated virus vector stimulated proliferation and significantly increased both type I and III collagen production in both primary human anterior cruciate ligament (ACL) fibroblast culture and *in situ* fibroblasts in the injured and non-injured ACL specimens. Apart from recombinant vectors, a particle-based FGF-2 delivery to wounds in mouse has also been shown to promote fibroblast proliferation and collagen deposition [27]. Altogether, PEMF has been demonstrated to increase proliferation and collagen deposition in both *in vivo* and *in vitro* models including diabetic wound models. Instead of directly acting on fibroblast proliferation and production of collagen by PEMF, the mechanisms may likely involve endothelial release of FGF-2 upon PEMF exposure. Then, the FGF-2 may act on the fibroblasts to promote both proliferation and collagen deposition possibly also through alpha-smooth muscle actin, nuclear factor kappa B [26] and vascular endothelial growth factor [27].

Another important growth factor in wound healing is transforming growth factor beta (TGFβ). PEMF enhances the activity of osteoblast and inhibit that of osteoclast [28] possibly through up-regulating the expression of TGFβ [28, 29] and downstream connective tissue growth factor (CTGF) [28]. While TGFβ and CTGF may play roles in the collagen deposition and maturation in healing, the effects of PEMF on soft tissues such as wounds may not be mediated through these growth factors as in bone tissue. Few studies showed PEMF could alter the level of TGFβ in wounds. Although TGFβ is well known to promote differentiation of fibroblast into myofibroblast, there is a lack of evidence that PEMF changes the level of TGFβ in diabetic wounds. As for other soft tissues, Jasti et al. [30] found that PEMF exerted no significant influences on the systemic expression of TGFβ in a rat tendinitis model. Another *in vivo* study of nerve lesion demonstrated no significant changes in the expression of either TGFβ or the downstream SMAD proteins after PEMF treatment [31]. Thus, there is no convincing evidence to support the involvement of TGFβ signalling on PEMF-mediated collagen deposition in wound healing to date. Future studies are warranted to investigate the effects of PEMF on the biomolecules causing changes in different extracellular matrices, including collagen and proteoglycans.

We postulate that the decreased material properties by PEMF might be because PEMF up-regulates and even prolongs collagen deposition, and interferes with the remodelling after complete re-epithelialization [32]. The paradoxical effects of the PEMF resemble very much the roles of calpains in wound healing [33]. Both PEMF and calpains might modulate collagen deposition [16], neo-vascular growth [14], alpha-smooth-muscle-actin-expressing myofibroblasts [15], and excessive collagen deposition. Future studies to explore the relationships between the effects of PEMF and the endogenous calpains are warranted. It is also noteworthy to confirm the potential increase of fibrosis, which may be related to the reduction in maximum stress, during the remodelling phase by the current PEMF protocol. Our findings demonstrated that there was a therapeutic window of PEMF parameters; various combinations of intensity and treatment period may produce different effects on the synthesis and remodelling of the extracellular matrix. Therefore, more research work is needed to determine a specific set of optimal PEMF parameters for treating diabetic wound healing in clinical settings. Multiple factors such as the severity of the wound, the stage of wound healing, possible infections or the patient’s medical history could be considered for the selection of appropriate PEMF parameters.

In conclusion, a 3-week exposure to PEMF of 10 mT and 25 Hz significantly increased energy absorption capacity and appeared to increase maximum load in the early proliferative phase, but decreased Young’s modulus in the remodelling phase of diabetic wounds. At the examined time points, the current PEMF protocols (both the 2-mT and 10-mT) thickened the wounds but reduced the maximum stress of diabetic wounds. PEMF may be effective to improve the tensile biomechanical strength of diabetic wounds during wound healing. However, further research work is needed to identify the optimal PEMF intensity and treatment time window before applying PEMF for treating diabetic wounds in clinical situations.

## Supporting information

S1 Table Number of wounds used in different groups at different time points. Wounds were randomly assigned into either 2-mT, 10-mT or sham PEMF groups. At each time point, they were randomly selected and harvested.
(DOCX)

S1 Data Raw data for Fig 2 describing the wound area (A) and percentage wound area (pA) of PEMF-treated (2 mT or 10 mT) and sham (0 mT) groups on post-wounding day 0, 3, 5, 7, 10, 14 and 21. Note that since wounds on day 14 and 21 were all closed (i.e. the A and pA are all 0), some observations were omitted.
(SAV)

S2 Data Raw data for Figs 3 and 4 describing the maximum load (MaxL), maximum stress (MaxStr), energy absorption capacity (energy), Young’s modulus (Young) and wound thickness (thickness) of wounds in PEMF-treated (2 mT or 10 mT) and sham (0 mT) groups on post-wounding day 3, 5, 7, 10, 14 and 21. (XLSX)
