# Correction: Effects of pulsed electromagnetic field (PEMF) on the tensile biomechanical properties of diabetic wounds at different phases of healing

**DOI:** 10.1371/journal.pone.0208475

**Published:** 2018-11-28

**Authors:** Harry M. C. Choi, Alex K. K. Cheung, Gabriel Y. F. Ng, Gladys L. Y. Cheing

The second author’s name is spelled incorrectly. The correct name is: Alex K.K. Cheung. The correct citation is: Choi HMC, Cheung AKK, Ng GYF, Cheing GLY (2018) Effects of pulsed electromagnetic field (PEMF) on the tensile biomechanical properties of diabetic wounds at different phases of healing. PLoS ONE 13(1): e0191074. doi:10.1371/journal.pone.0191074
