# Supplementary material for: Effects of pulsed electromagnetic field (PEMF) on the tensile biomechanical properties of diabetic wounds at different phases of healing
Source: PLoS One. 2018 Jan 11;13(1):e0191074. doi: 10.1371/journal.pone.0191074 (PMC5764361; doi:10.1371/journal.pone.0191074)
Supplement: S1 Table — Wounds were randomly assigned into either 2-mT, 10-mT or sham PEMF groups. At each time point, they were randomly selected and harvested. (DOCX) [file pone.0191074.s001.docx]

S1 Table: Number of wounds used in different groups at different time points.

| Post-wounding day | | 3 | 5 | 7 | 10 | 14 | 21 | Total |
| --- | --- | --- | --- | --- | --- | --- | --- | --- |
| PEMF groups | 2 mT | 14 | 16 | 6 | 6 | 10 | 12 | 64 |
|  | 10 mT | 9 | 9 | 10 | 10 | 15 | 17 | 70 |
| Sham group | | 12 | 13 | 8 | 12 | 21 | 15 | 81 |
| Total | | 35 | 38 | 24 | 28 | 46 | 44 | 215 |
